# Severity of neuromeningeal tuberculosis in Morocco: a critical evaluation of epidemiological trends and treatment efficacy

**DOI:** 10.11604/pamj.2024.47.216.42573

**Published:** 2024-04-29

**Authors:** Fadia Bejja, Hinde Hami, Fatiha Aboulhoda, Fatine Hadrya, Abdelrhani Mokhtari, Abdelmajid Soulaymani

**Affiliations:** 1 Laboratory of Biology and Health, Faculty of Science, Ibn Tofail University, Kenitra, Morocco,; 2 University Hassan First of Settat, Higher Institute of Health Sciences, Health Sciences and Technologies Laboratory, Settat, Morocco

**Keywords:** Neuromeningeal tuberculosis, epidemiology, public health, clinical manifestations, treatment outcomes

## Abstract

**Introduction:**

neuromeningeal tuberculosis (NMT) is a significant public health challenge in Morocco because of its acute severity and high mortality rates. This study aims to comprehensively evaluate the epidemiological, clinical, therapeutic, and disease progression characteristics of NMT in the Kenitra province.

**Methods:**

a retrospective analysis was conducted on the medical records of patients diagnosed with NMT at the Diagnostic Center of Tuberculosis and Respiratory Diseases in Kenitra from 2014 to 2017.

**Results:**

among the 33 identified NMT cases, predominantly males (57.6%) were affected, with an age range of 4-76 years and a median age of 25 years. Extrapulmonary manifestations were prevalent, constituting 78.8% (n=26) of all cases, with meningeal localization in 45.5% (n=15) of confirmed cases. Furthermore, 9.1% (n=3) of cases were associated with unconfirmed cerebral tuberculosis (TB), and 12% (n=4) exhibited miliary TB. Familial transmission and comorbidities were identified as significant factors in disease progression. More than half of the patients received standardized antibacillary treatment during hospitalization, which lasted between 9 and 12 months. Treatment outcomes were generally positive (73%), but a 12% case fatality rate and 15% loss to follow-up were observed.

**Conclusion:**

this study highlights the complex clinical and public health challenges posed by NMT in Morocco. It emphasizes the need for improved health strategies that not only increase public awareness but also enhance the accessibility and quality of diagnostic services and treatment options.

## Introduction

Tuberculosis (TB) is a significant global public health concern. According to the World Health Organization (WHO), an alarming number of 10.6 million individuals contracted TB worldwide in 2021, affecting 6 million men, 3.4 million women, and 1.2 million children [1]. Tuberculosis (TB) causes a significant burden of morbidity and mortality, with 1.6 million individuals dying from the disease in 2021, including 187,000 people co-infected with HIV [2]. Tuberculosis (TB) can manifest in various forms, intrapulmonary and extrapulmonary, affecting other organs beyond the lungs. These include a range of variants such as meningeal/cerebral, osteoarticular, lymphatic, pleural, renal, pericardial, peritoneal, and genital presentations [3,4]. Among these extrapulmonary forms, Neuromeningeal Tuberculosis (NMT) is considered the most severe manifestation of mycobacterium TB infection [1,5]. Although TB primarily affects the lungs, it can also cause extrapulmonary manifestations, accounting for 10 - 42% of cases [6,7]. This presents diagnostic challenges and requires prolonged treatment, potentially leading to long-term consequences. However, early detection and careful treatment can lead to a full recovery. Neuromeningeal tuberculosis is frequently observed in HIV-positive patients and children under 2 years of age, causing a medical emergency where any delay in diagnosis or treatment could raise the risk of death or irreversible nerve damage [8]. Multiple screening methods are used to combat TB, including obtaining medical histories, conducting clinical and laboratory assessments, performing chest radiography, administering Tuberculin Skin Tests (TST) or Interferon Gamma Release Assays (IGRA), culturing pathological samples, and testing for anti-TB drug resistance using online reverse hybridization (ORI) techniques. The molecular biology technique (Xpert MTB/RIF) is specifically used to detect Mycobacterium TB [4,9]. Identifying NMT is daunting because of its highly diverse clinical presentation and non-specific radiological signs, leading to delayed treatment initiation [10].

Morocco reported over 35,000 cases of TB and 2,974 deaths in 2019, with an incidence rate of over 97 per 100,000 individuals and a case fatality rate (CFR) of 8% [11]. The Moroccan Ministry of Health has launched an extended strategic plan for TB prevention and control, covering the period 2021 - 2023. The aim is to address the pressing public health concern and reach a 60% reduction in TB-related fatalities by 2023 compared to deaths recorded in 2015 [11]. The Rabat-Salé-Kenitra region showed the second-highest incidence rate in 2019, accounting for 18% of the total cases with an incidence rate of 98 cases per 100,000 individuals [12]. Our study aimed to provide a comprehensive analysis of the epidemiological, clinical, therapeutic, and disease progression characteristics of patients with NMT in the Kenitra province, Morocco.

## Methods

**Study design:** this study used a cross-sectional retrospective analysis to assess the epidemiological, clinical, therapeutic, and disease progression characteristics of NMT in the Kenitra province.

**Study setting and population:** the Kenitra province is situated in the northwest of Morocco, approximately 40 km from the capital city of Rabat. It covers an area of 3,052 km^2^and has significant demographic and geographical diversity. The population, according to the 2014 census, is 1,061,435, with 606,993 individuals residing in urban areas and 454,442 in rural areas, resulting in a population density of approximately 348 inhabitants per square mile. Administratively, the province is divided into five districts (circles) and twenty-three communes.

The region´s proximity to the Atlantic Ocean and fertile land has fostered significant economic growth, particularly in the automotive manufacturing sector and agriculture, with notable production of cereals, vegetables, and citrus fruits. Economic development in the province is juxtaposed with the inconsistent quality of medical infrastructure, particularly in rural areas, which affects the surveillance and treatment of TB.

The Diagnostic Center of Tuberculosis and Respiratory Diseases (DCTRD) in Kenitra is the central site for this study and is responsible for collecting TB data and conducting epidemiological surveillance. Data were extracted from TB patient reporting registers, including all patients diagnosed and treated for the condition within the province. The limitations of the variables available and the potential for sample bias due to the single-center nature of data collection are acknowledged. However, the data are still considered to provide valuable insights for the field.

**Variables:** this study analyzed several explanatory variables, including gender, age, place of residence, and employment status. The clinical form of TB was classified into pulmonary or extrapulmonary types, and patient engagement with treatment was monitored, noting whether it was in the initiation or continuation phase. Bacteriological confirmation of TB was included and ascertained through laboratory assessment and clinical diagnosis. HIV status was determined serologically and categorized as positive, negative, or indeterminate. Comorbidities were also recorded. Specific symptomatology related to NMT and other relevant clinical data were comprehensively recorded.

Outcomes were evaluated based on therapeutic response, including successful treatment, unsuccessful treatment, and mortality during the treatment period. The operational definitions for these outcomes were aligned with the WHO revised standards as of 2016 [12], ensuring methodological rigor and global comparability of the results.

**Data resource and measurement:** data extraction and processing: data were extracted from patient records with meticulous attention to detail using the Tuberculosis Patient Notification Register as the primary source for data verification and extraction. The data were manually entered into Microsoft Excel with rigorous scrutiny to ensure data consistency.

For quantitative variables, we employed a manual discretization process using thresholds recognized by the scientific community to ensure comparability with existing research.

**Data collection:** this study was conducted through a retrospective analysis of TB patient records in the Kenitra province from 2014 to 2017, focusing on individuals diagnosed with NMT. A comprehensive set of data was extracted from the patient´s medical records, including sociodemographic factors such as age, gender, and place of residence, as well as other risk factors for TB. To elucidate disease progression, clinical parameters such as symptomatology and observed progression in patients were documented.

**Sample size:** this study presents a comprehensive retrospective analysis of all recorded cases of NMT in the Kenitra province, encompassing 33 patients who received treatment and follow-up from January 1, 2014, to December 31, 2017.

**Data analysis:** statistical analyses were conducted using SPSS software (trial version) to examine the trends and characteristics of NMT in Kenitra from 2014 to 2017. This commenced with a descriptive analysis, which calculated means and standard deviations for quantitative variables and frequencies for qualitative variables to outline the cohort´s profile. Linear regression analysis was used to determine the temporal trend of NMT cases, providing insights into the effects of public health interventions and disease progression.

Associations between sociodemographic variables and pivotal health outcomes were also assessed, including treatment success and mortality. The analysis examined the influence of age, gender, and residential status on epidemiological patterns.

The methodological approach chosen for this study included the computation of annual CFR. This approach was selected for its robustness and relevance to the single-center study design, ensuring statistically robust and contextually significant findings.

**Ethical consideration:** ethical compliance was a priority. We obtained all necessary authorizations before beginning data collection, ensuring adherence to confidentiality and data subject anonymity standards.

## Results

**Figure 1:**
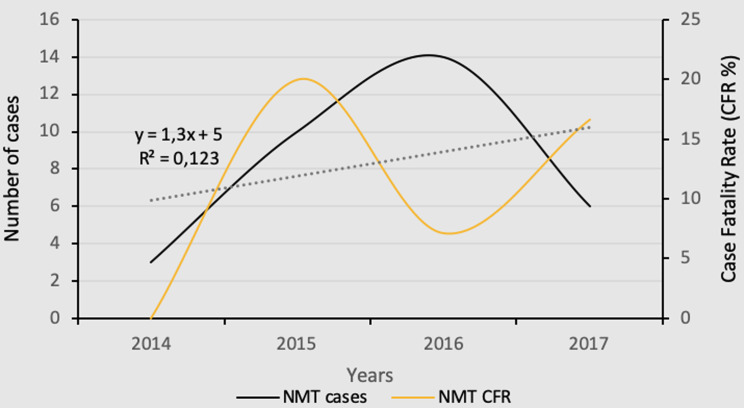
annual trends in neuromeningeal tuberculosis cases and associated case-fatality rates

**Sociodemographic characteristics of patients:** a comprehensive analysis of the medical records of 33 patients diagnosed with NMT during the study period was conducted. The incidence and mortality rates associated with NMT demonstrated temporal fluctuations characterized by variations in the number of cases and associated deaths, as shown in Figure 1. Between 2014 and 2015, there was a correlated increase in the number of diagnosed cases and CFR. However, this trend was subsequently reversed, with an increase in cases correlating with a decrease in CFR and vice versa. Furthermore, it is worth noting that the time frame, measured in years, accounts for approximately 12.3% of the variations observed in the NMT case count, highlighting the impact of other variables or medical interventions.

Of this cohort, 19 individuals (57.6%) were male, resulting in a male-to-female sex ratio of 1.37. The mean age of the patients was 32.94 ± 19.35 years, with ages ranging from 4 to 76 years. Of the total patients, 19 were unmarried, as shown in Table 1.

**Table 1:** distribution of neuromeningeal tuberculosis cases by category

Category	NMT cases	NMT frequency (%)
**Gender**		
Male	19	57.6
Female	14	42.4
**Age group**		
0-14 years	5	15.2
15-24 years	11	33.3
25-64 years	14	42.4
65 years and older	3	9.1
**Marital status**		
Married	14	42.4
Unmarried	19	57.6
**Occupation**		
Unemployed	13	39.4
Student	12	36.4
Self-employed	8	24.2
**Place of residence**		
Rural	18	54.5
Urban	15	45.5

NMT: neuromeningeal tuberculosis

Regarding occupational status, 39.4% of the patients were jobless, 24.2% were self-employed, and 36.4% were students. Moreover, 54.5% of the population came from rural areas.

The patients´ household size had a mean of 6.28 ± 2.37, with a range of 2 to 13 individuals. The examination conducted by the medical providers showed that 48.5% (n=16) lacked insurance, while 24.2% were beneficiaries of the National Fund for Social Welfare Organizations, *Caisse Nationale des Organismes de Prevoyance Sociale* (*CNOPS*), 12.1% (n=4) were members of the National Social Security Fund (*CNSS*), and 15.2% (n=5) received benefits from the Medical Assistance Plan.

### Clinical features

**Figure 2:**
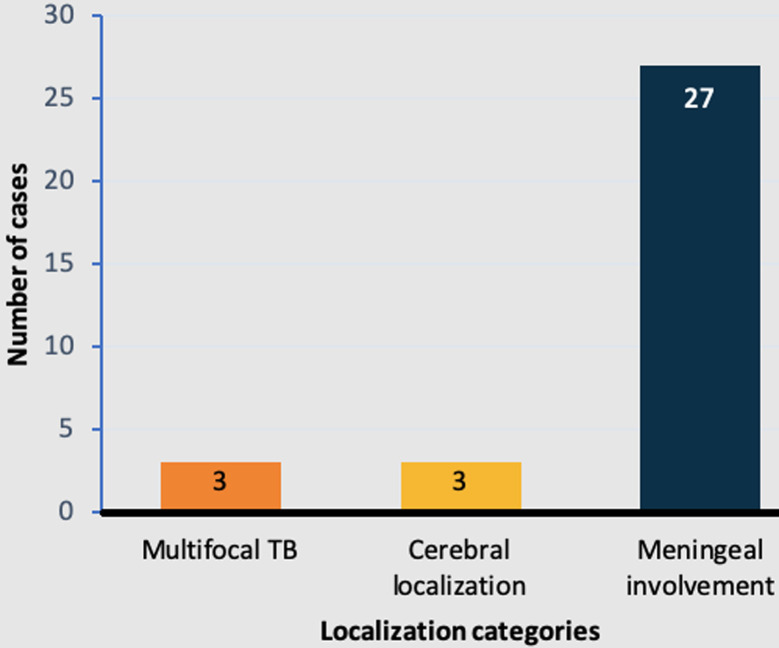
distribution of patients with neuromeningeal tuberculosis based on localization

**Meningeal involvement and cerebral localization:** meningeal involvement was observed in 82% (n=27) of the cases. Out of this subset, 56% (n=15) were confirmed cases. Cerebral localization was observed in 9% (n=3) of the cases, and multifocal TB was present in 9% (n=3) of the cases (Figure 2).

**Figure 3:**
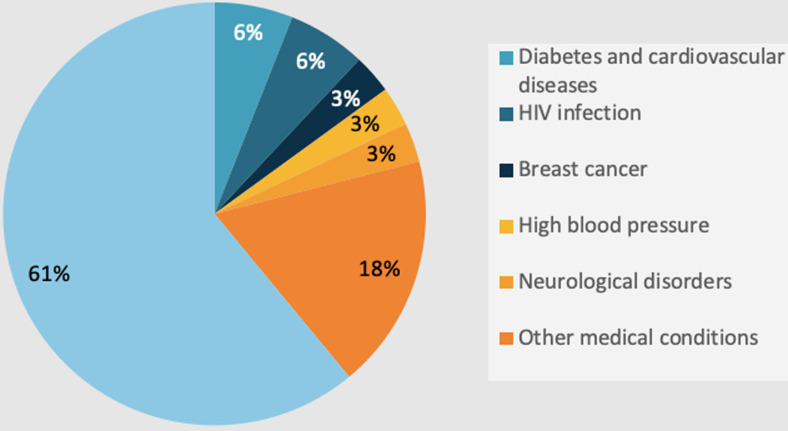
medical history of the study participants

**Medical history and tuberculosis infection:** the medical records of the patients indicated the existence of specific conditions in 21.2% of the cases (Figure 3). These conditions included one case of breast cancer in a woman, two cases of comorbidities of diabetes and cardiopathy in women, one case of hypertension in a man, two cases of TB and HIV co-infections in women, and one case of a neurological disorder in a woman. TB transmission was detected in 9.1% of patients (n=3), whereas the remaining patients did not display any symptoms of it (n=30). All three infected individuals were male students, and their infection was attributed to familial ties.

**Clinical and biological examination:** of the patient cohort, 60.6% (n=20) were hospitalized, comprising 11 females and 9 males. Moreover, 93.9% of patients visited DCTRD in Kenitra for the first time (new cases). Symptom onset was characterized as gradual or progressive. The most common clinical signs were fever (45.45%), weight loss (33.33%), and night sweats (30.30%). Neurological symptoms included neck stiffness (42.42%), headaches (36.36%), and altered consciousness. Rare clinical signs with frequencies below 20% included persistent cough, vomiting, and paralysis of the cranial nerves. Cerebrospinal fluid (CSF) analysis was performed on 11 patients (33%), two of whom showed hyperproteinorachia. In addition, 12.1% of the patients underwent thoracic radiological testing to rule out potential TB associations.

**Treatment and outcome:** all patients adhered to the antibacillary treatment guidelines of the National Tuberculosis Control Program and WHO recommendations. The duration of treatment varied: 76% of patients underwent a nine-month regimen (2RHZE/7RH), 12% received a twelve-month regimen (2RHZE/10RH), 9% of children were treated with a nine-month regimen (2RHZ/7RH), and 3% followed a twelve-month regimen (2RHZ/10RH). The functional prognosis of the reference regimen depended on the daily intake of rifampicin and isoniazid for seven to nine months, with supplementation of the intensive two-month phase with pyrazinamide and ethambutol for adults (2RHZE/7RH). The mean time between symptom onset and treatment initiation was 20.42 days (minimum 1 - maximum 71), while the mean treatment duration was 216 days, ranging from 0 to 377 days.

Corticosteroids have shown the potential to reduce mortality in cases of meningeal TB [13]. Among our cohort, 8 patients (24.2%) received corticosteroids, including one patient with breast cancer, one with neurological disease, and 3 with miliary TB. All 8 patients who received corticosteroids were admitted to the hospital. Seventy-three percent of the patients who successfully completed the 9- or 12-month treatment experienced positive therapeutic outcomes, whereas 15% were lost to follow-up. According to the data collected, four patients died, resulting in a CFR of 12.12%. Of the deceased, 33% were children, and they survived for 1 week (1 case), 2 weeks (1 case), and 8 weeks (2 cases) after treatment initiation. An infant, a child, and two adults were affected by these fatalities. Two of the deceased patients exhibited consciousness disorders (coma), and one died from fever and weight loss. Three cases exhibited sequelae following NMT. One patient had strabismus, another had hydrocephalus, and the third had paralysis.

## Discussion

Tuberculosis (TB) remains a significant infectious disease that can be managed through early detection, timely medical intervention, and consistent adherence to treatment, particularly in severe cases. Extrapulmonary manifestations, which occur in 10%-42% of TB cases, affect various body parts such as the lymph nodes, bones, nervous system, skin, and urinary system [11].

This study collected a total of 33 cases of NMT between 2014 and 2017. An investigation study conducted in Casablanca documented 52 cases of tuberculous meningitis from 2011 to 2014 [5]. Furthermore, a study conducted in Rabat on TB patients revealed only one case with neuromeningeal symptoms [14]. Moreover, a study involving 21 hospitalized patients from January 2002 to December 2016 was conducted in Meknes, which provided further insights [15].

According to a 2020 study conducted in the Congo, NMT cases exhibited a significant male predominance of 65% [16]. Brain lesions related to TB have a positive prognosis, with less than 3% of cases resulting in mortality and around 4% causing complications [13]. However, although brain lesions showed positive outcomes, the overall prognosis remained unfavorable because of high mortality rates, particularly among pediatric patients. Mortality rates as high as 30% have been reported. Our study aligned with this finding, reporting a mortality rate of 33% [14].

According to a 2012 study conducted in Madagascar, central nervous system TB accounted for 5%-15% of extrapulmonary TB cases and 1%-1.3% of all TB cases [17]. Immune-compromising individuals have a higher susceptibility to TB [18]. TB is commonly associated with various comorbidities, such as diabetes, TB/HIV, and risk factors, including smoking, alcohol abuse, drug addiction, and mental disorders [19].

The occurrence of TB is still high and worsened by co-infection with HIV [20]. According to the WHO guidelines, the initial TB treatment regimen involves administering quadruple therapy followed by dual therapy continuation in the secondary phase [21]. Typically, corticosteroid therapy is administered alongside antibacterial treatment to expedite the regression of tuberculomas and decrease the chances of paradoxical expansion during the initial weeks of treatment, known as the Jarisch-Herxheimer reaction phenomenon [20].

As specified by Bazin *et al*. risk factors usually include geographical context, household size, and latency period for symptom manifestation [22]. Almost 30% of TB cases will have an impact on the central nervous system [23]. Gradual clinical deterioration usually accompanies meningitis caused by TB, requiring hospitalization and leading to advanced stages at admission and diagnostic delays [8,24].

**Limitations:** this study has a retrospective design, which may introduce biases due to its reliance on the accuracy and completeness of existing medical records. Variability in data recording processes can lead to discrepancies, which may affect the reliability and validity of the findings. Retrospective analyses are particularly susceptible to information bias, which can distort the study results and lead to underestimation or overestimation of the effects. Furthermore, it is possible that the existing medical records do not cover all the complex factors that affect TB morbidity and mortality rates, including lifestyle factors such as substance abuse or alcohol consumption. Therefore, future research should consider including a wider range of sociocultural and behavioral variables to gain a more nuanced understanding of the disease´s impact. While the dataset is comprehensive and represents a significant number of NMT cases within the Kenitra province, it is important to exercise caution when extrapolating these findings to other contexts due to the geographically specific nature of the data collected.

## Conclusion

This study was conducted at the Diagnostic Center of Tuberculosis and Respiratory Diseases in the Kenitra province. It aimed to discern the epidemiological trends of NMT over a span of four years. Analysis of sociodemographic variables revealed temporal patterns in disease incidence and mortality. It also emphasized the urgent need for clinical responses because of the prevalence of meningeal involvement. Although therapeutic protocols are effective, post-treatment patient retention remains a challenge, highlighting the need for systemic enhancements in healthcare follow-up practices. This study emphasizes the importance of culturally and behaviorally appropriate interventions in the Moroccan context. It proposes a foundational perspective for future inquiries aimed at amplifying intervention strategies and ultimately alleviating the burden of severe TB manifestation.

### What is known about this topic

- *Tuberculosis, particularly its neuromeningeal manifestation, poses significant diagnostic challenges and has a considerable impact on morbidity and mortality, underscoring an imperative to address this public health emergency in Morocco;*
- *Despite the acknowledgment by Moroccan health authorities that tuberculosis is a serious health concern, there is a lack of scientific data on the disease´s severe manifestations and associated risk factors, particularly in the Kenitra province*.

### What this study adds

- *This study provides the first comprehensive epidemiological analysis of neuromeningeal tuberculosis in the Kenitra province, revealing significant temporal trends in incidence and mortality, as well as the effects of sociodemographic factors on disease progression;*
- *The study identifies key variables that are empirically correlated with neuromeningeal tuberculosis prognosis, including age, gender, rural residence, patterns of disseminated tuberculosis, and the prevalence of comorbidities. The study also examines the high mortality rates in cases presenting with miliary tuberculosis, which paradoxically exhibit higher rates of remission*.
